# Emergency management: acute endophthalmitis

**Published:** 2018-11-09

**Authors:** Nuwan Niyadurupola

**Affiliations:** 1 Consultant Ophthalmic Surgeon & Honorary Senior Lecturer: Ophthalmology Department, The Norfolk and Norwich University Hospital NHS Trust, Norwich, UK.

**Endophthalmitis can have devastating consequences for a patient's eye and vision. Prompt recognition and urgent treatment are vital.**

**Figure:**
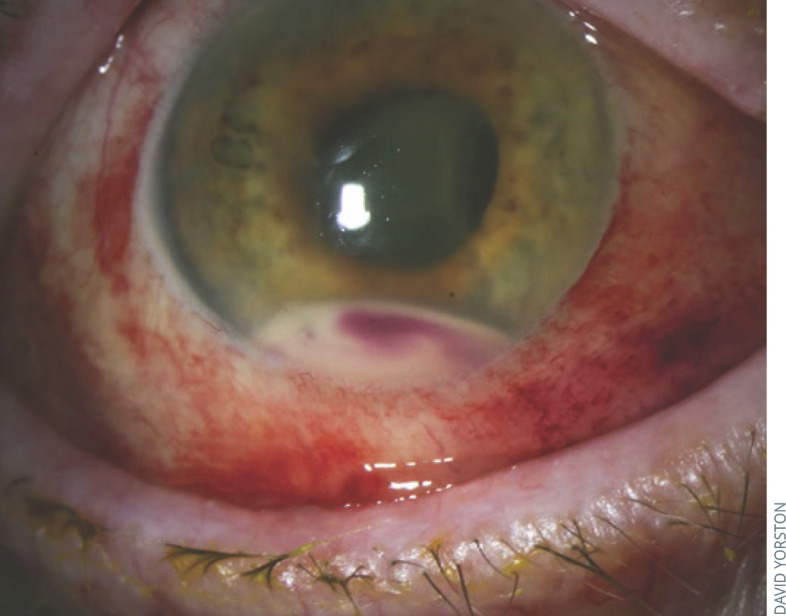
Endophthalmitis. The eyelids are swollen, the eye is red and a hypopyon is clearly visible.

## How to recognise endophthalmitis

1. Suspect endophthalmitis if **any** of the following symptoms or clinical signs are present, particularly if there is a previous history of surgery, intravitreal injection or penetrating trauma:
  - Blurred vision
  - Pain
  - Red eye
  - Hypopyon
  - Vitreous opacities
  - Swollen eyelids
  - Poor red reflex
2. Perform B-scan ultrasonography (if available) to check for vitritis or retinal detachment.
3. Do not try to treat with a course of corticosteroids first – this will delay treatment and may result in losing the eye.

## Protocol: How to respond to the condition

**Do not delay! Treat as a medical emergency**

### Within 1 hour

- Perform an intravitreal tap or vitrectomy through the pars plana (see panel). Collect samples of vitreous for Gram stain and culture. A vitrectomy may be indicated if the patient has perception of light only. *However, if a delay is likely before a vitrectomy can be performed, it is advisable to perform a vitreous tap and inject intravitreal antibiotics for more rapid treatment*
- Immediately following the intravitreal tap, inject antibiotics into the vitreous (see panel)
- After injecting intravitreal antibiotics, use a different syringe and a 30-gauge needle to inject preservative-free dexamethasone (400 μg in 0.1 ml) into the vitreous.

**Technique: How to do an intravitreal tap**

- Use aseptic technique with drape
- Instil topical antibiotics and povidone iodine 5%
- Administer subconjunctival or sub-Tenon's anaesthetic
- Insert a 23-gauge or 25-gauge needle 4 mm (phakic eyes) or 3.5 mm (pseudoaphakic/aphakic eyes) behind the limbus into the middle of the vitreous cavity, pointing at the optic disc (approx 7–8 mm deep) and aim to aspirate 0.3–0.5 ml of vitreous fluid.

**Antibiotics**

1st choice:

- Vancomycin 1 mg in 0.1 ml and
- Ceftazidime 2 mg in 0.1 ml

**OR**

2nd choice:

- Amikacin 400 μg in 0.1 ml and
- Ceftazidime 2 mg in 0.1 ml

**Note: Use a new syringe and a new 30-gauge needle for each drug. Do not mix drugs together in the same syringe.**

### Then

- Consider adjunctive systemic therapy, with the same antibiotics as those used intravitreally, for 48 hours. This will maintain higher levels within the posterior segment of the eye. If systemic antibiotics are not available, topical antibiotics are better than nothing
- Monitor the patient carefully
- Use the response to treatment and the results of Gram stain and culture to determine whether further intravitreal antibiotic therapy is required.

## Preparing for the emergency

An endophthalmitis kit should be accessible in every practice where postoperative patients are seen. This is essential for the prompt diagnosis and treatment of endophthalmitis. Include instructions for preparing the antibiotics (see p. 69).

### Equipment for preparation of patient

- Tetracaine (anaesthetic) drops
- Povidone iodine
- Drape
- Speculum

### Equipment for sub-Tenon's anaesthetic injection

- 10 ml 2% lidocaine
- 10 ml syringe
- Sub-Tenon's cannula
- Westcott scissors

### Equipment for vitreous biopsy/tap

- 23- gauge or 25-gauge needle
- 5 ml syringe
- Calipers

### Equipment for preparation of antibiotic injections

- 1 vial of 500 mg vancomycin or 1 vial of 500 mg (250 mg/ml) amikacin
- 1 vial of 500 mg ceftazidime
- 3 × 10 ml sodium chloride 0.9% injection (saline)
- 4 × 10 ml syringe
- 2 × 5 ml syringe
- 2 × 1 ml syringe
- 1 × sterile galley pot (for amikacin)
- 6 × 21-gauge needles for preparation of antibiotics
- 2 × 30-gauge needles for intravitreal injection

## Instructions for preparation of antibiotic injections

### Vancomycin 1 mg/0.1 ml

- Reconstitute 500 mg vial with 10 ml saline
- Withdraw all 10 ml into 10 ml syringe
- Inject 2 ml of this solution back into vial
- Add 8 ml saline into vial to make up to 10 ml (10 mg/ml)
- Use 1 ml syringe to draw 0.1 ml of this solution (1 mg/0.1 ml)

### Amikacin 400 μg/0.1 ml

- Use 10 ml syringe to withdraw 1.6 ml of amikacin (250 mg/ml)
- Make up to 10 ml in the syringe with saline
- Discard 9 ml from syringe and make the remaining 1 ml up to 10 ml (in the syringe) by adding saline
- Transfer the solution into a sterile galley pot and use 1 ml syringe to draw 0.1 ml of this solution (400 μg/0.1 ml)

### Ceftazidime 2 mg/0.1 ml

- Reconstitute 500 mg vial with 10 ml saline
- Withdraw all 10 ml into a 10 ml syringe
